# Rhizobacterial Community Assembly Patterns Vary Between Crop Species

**DOI:** 10.3389/fmicb.2019.00581

**Published:** 2019-04-04

**Authors:** Andrew Matthews, Sarah Pierce, Helen Hipperson, Ben Raymond

**Affiliations:** ^1^College of Life and Environmental Sciences, University of Exeter, Penryn, United Kingdom; ^2^Department of Life Sciences, Imperial College London, Ascot, United Kingdom; ^3^School of Life Sciences, University of Nottingham, Nottingham, United Kingdom; ^4^Department of Animal and Plant Sciences, P3 Institute for Plant and Soil Biology, The University of Sheffield, Sheffield, United Kingdom

**Keywords:** rhizobacteria, 16s r RNA gene sequencing, community structure, host colonization, PGPR (Plant Growth Promoting Rhizobacteria)

## Abstract

Currently our limited understanding of crop rhizosphere community assembly hinders attempts to manipulate it beneficially. Variation in root communities has been attributed to plant host effects, soil type, and plant condition, but it is hard to disentangle the relative importance of soil and host without experimental manipulation. To examine the effects of soil origin and host plant on root associated bacterial communities we experimentally manipulated four crop species in split-plot mesocosms and surveyed variation in bacterial diversity by Illumina amplicon sequencing. Overall, plant species had a greater impact than soil type on community composition. While plant species associated with different Operational Taxonomic Units (OTUs) in different soils, plants tended to recruit bacteria from similar, higher order, taxonomic groups in different soils. However, the effect of soil on root-associated communities varied between crop species: Onion had a relatively invariant bacterial community while other species (maize and pea) had a more variable community structure. Dynamic communities could result from environment specific recruitment, differential bacterial colonization or reflect broader symbiont host range; while invariant community assembly implies tighter evolutionary or ecological interactions between plants and root-associated bacteria. Irrespective of mechanism, it appears both communities and community assembly rules vary between crop species.

## Introduction

Plants coexist with complex microbial communities both in above-ground organs (the phyllosphere) and below-ground (the rhizosphere), the rhizosphere including both the inside of the root tissue and the soil immediately adjacent to and under the influence of the root system. Hiltner, as early as 1901, predicted that the resistance of plants toward pathogens is dependent on the composition of a “plant microflora” and that root exudates of different plants could support development of different microbial communities (Hartmann et al., 2007). The signaling interactions and host association patterns between model plants and some of their specialized pathogens and mutualists are well-documented (Philippot et al., 2013). Moreover, a wide range of physiological benefits for plants may result from the association with bacteria; benefits that include nutrient acquisition, enhanced stress tolerance, host immune regulation, and protection against soil borne pathogens and phytophagous insects (Pineda et al., 2010; Berendsen et al., 2012; Mendes et al., 2013; Turner et al., 2013a; Bakker et al., 2014; Cook et al., 2015). Microbiome effects on host immunity are thought to be particularly crucial. Despite their importance, we are at an early stage of understanding the factors that shape the composition of plant microbial communities. If we understood rhizosphere community assembly then this process would potentially be more predictable or controllable (Ho et al., 2013; Krause et al., 2014); improving our ability to selectively promote species with direct benefits for crop growth or to selectively inhibit pathogens.

Root-associated microorganisms are mainly recruited from the surrounding soil. Unsurprisingly, these communities are strongly influenced by the composition of the reservoir of soil microbes (Bulgarelli et al., 2012; Lundberg et al., 2012; Schlaeppi et al., 2014). In tree rhizospheres, in particular, fungal community composition is more *influenced by soil rather* than plant species (Bonito et al., 2014). However, plant taxa can significantly influence the formation of root-inhabiting bacterial assemblages when different cultivars, species or distinct genotypes of plants are grown in the same soil (Manter et al., 2010; Bouffaud et al., 2014; Ofek et al., 2014). Plant specific rhizodeposits are one mechanism shaping bacterial community shifts from soil to host-adapted communities, with reduced diversity and increased abundance of select core taxa (Bulgarelli et al., 2012; Lundberg et al., 2012). Plants may also modulate their rhizosphere microbiome so that different plant species promote or enrich a particular set of microbes (Haichar et al., 2008; Turner et al., 2013b; Ofek-Lalzar et al., 2014). Plant evolutionary history appears to be important here: with an increase in the phylogenetic distance between plant species, differences in microbial assemblages also increase (Wieland et al., 2001; Bouffaud et al., 2014). Not only different plant species, but also different genotypes of the same species, may differ in their rhizosphere microbiome (Inceoglu et al., 2011; Weinert et al., 2011; Peiffer et al., 2013).

Initial studies to characterize plant-associated communities relied on cultivation-based methods. Although culture-dependent studies can make important conclusions about specific, readily isolated microbes (Bakker et al., 2013), they are biased in the taxa they identify and drastically limit community diversity estimates. Recent consensus emerging from next generation sequencing studies is that four major bacterial phyla (Proteobacteria, Actinobacteria, Bacteroidetes, and Firmicutes) make up the vast majority of members of root associated bacterial communities (Hacquard et al., 2015).

As a consequence of this repeatedly observed broad scale structure, bacterial community assembly in the rhizosphere has been viewed as a deterministic and tightly controlled process (Bulgarelli et al., 2013; Haney and Ausubel, 2015). However, experimental approaches designed to test the many factors at play in community assembly are scarce, and community assembly models are still largely untested. Understanding the factors that shape or limit community assembly in the rhizosphere may be of applied importance. First, altered communities of rhizosphere bacteria have a role in plant invasiveness (Coats and Rumpho, 2014; Gundale et al., 2014). Second, despite being a rapidly developing industry, the application of rhizobacteria as plant growth promoters (PGPR) can have variable effects in the field (du Jardin, 2015). One of the possible causes of these variable results is that we do not fully appreciate whether community assembly patterns (local adaptation or prior occupation of host niches) are likely to determine if applied PGPR have an opportunity to interact with hosts at all.

Currently both hosts and soils are implicated as key determinants of rhizosphere bacterial communities, yet few studies have explicitly tested their relative importance in shaping whole bacterial communities or fully controlled for the effects of sampling site. The aim of this study was to compare the effects of soil and plant host on bacterial community assembly in the rhizosphere. We used a replicated split plot, glasshouse mesocosm design, with two soil treatments, and four taxonomically distinct host treatments. These generated bacterial samples from two soil types and roots of different hosts grown under controlled conditions, which were characterized using an in-depth next generation metagenomic amplicon sequencing approach. We then compared bacterial diversity and community composition across species and soils to assess their effect on the rhizosphere microbiota.

## Materials and Methods

### Soil Sampling

Grassland and woodland soils were used in these experiments because they represented qualitatively different soil environments, characterized by different associated vegetation communities–namely a mesotrophic grassland and mixed deciduous oak/birch woodland (Crawley, 2005). Woodland soil samples from Silwood Park; Nash's Copse (Lat: 51° 24′ 49.8024″ Long:−0° 38′ 48.357″) and grassland soil samples from Nash's Field (Lat: 51° 24′ 44.5400″ Long: −0° 38′ 41.357″) were taken on the 9th of April 2013. Three trenches 0.3 m^2^ by 1 m long were dug at 10 m intervals in two parallel transects 250 m apart, to remove approximately 40 L of soil and vegetation per trench, giving 120 L in total per soil treatment. Soil screening and decanting soil into pots occurred the following day. Detailed site descriptions and methods for soil screening and pot mesocosm set up are supplied in Data Sheet 1.

### Glasshouse Culture Regime and Sampling

Four varieties of organic untreated seed were supplied by Moles Seed (UK) Ltd: *Allium fistulosum* L. var Ishikura (onion); *Pisum sativum* L. var Twinkle (pea); *Solanum lycopersicum* L. var MicroTom (tomato); *Zea mays* L. var Minipop (sweet corn). Seeds were surface sterilized and incubated at 24°C in 30 mL sterile water for 2 days before sowing. In addition seed surface sterilization checking plates were set up to validate this process Data Sheet 1. Twenty onion, six pea, ten tomato, and six sweet corn seeds were sown in each pot at approximately 2 cm depth and spacing. Single species monocultures were grown as nested host treatments in a quarter of a 7.5 L terracotta pot. Pots were imbedded in wet sand in 13 L buckets, in order to minimize water stress, and divided into quarters by plastic dividers (Correx, Cricklade, Wiltshire, UK).

Mesocosms were cultured for between 60 and 86 days in total. The glasshouse simulated 16 h light cycles from 06:00 to 22:00 with 400 W full spectrum lights suspended 1.5 m above the growing plants, and at 1.5 m spacing along the benches. Artificial lighting cut out when ambient light exceeded 450 lux for more than 5 min. The initial 14 days of culture, during seed germination and establishment, used direct surface watering with sterile water. Further irrigation used 500 ml tap water applied indirectly to sand every 1–2 days and occasional direct sterile surface watering. Pots were randomly moved within the glasshouse every 10–12 days. Heating and passive cooling via ceiling vents maintained the temperature between 20 and 27°C.

Pots, from both soil treatments, were randomly sampled in pairs between 2/6/13 and 3/7/13. After sampling the following host traits were measured: %germination, fresh aboveground biomass, dry aboveground biomass, fresh belowground biomass, Pea nodulation frequency. Dry belowground biomass was not recorded as within a pot whole rootstocks from all the surviving individuals were washed thoroughly, pooled by host, and destructively sampled for bacterial extraction.

### Soil Moisture and Nutrient Profiling

In order to test for differences in soil characteristics between soil treatments we quantified available and total nutrients. Specifically we assessed available free nitrogen (nitrate/nitrite and ammonia) available phosphate, total N & P, C:N, and soil moisture. Detailed methods can be found in the Supplementary Material.

### Bacterial Cell Enrichment

Bacterial sampling methods from whole rootstocks followed protocols established to reduce the presence of plastid DNA (Ikeda et al., 2009, 2010). Soil was removed by shaking and washing the rootstock in an excess volume of sterile H_2_O. Rootstocks were weighed and homogenized in 120 ml of pre-chilled bacterial cell enrichment (BCE) buffer (1L H_2_O, 50 ml 1M Tris, 10 ml Triton X100, 2 ml β-mercaptoethanol) using a blender at high speed for 3 min. The homogenate was filtered through a layer of sterilized Miracloth and centrifuged at 1,800 rpm for 5 min at 10°C. The supernatant was further centrifuged at 4,000 rpm for 20 min and the pellet re-suspended in 5 ml of BCE buffer. Samples were filtered into one 15 ml falcon tube through 4 layers of sterilized Kimwipe. Filtrates were then centrifuged at 4,000 rpm for 10 min at 10°C. The supernatant discarded and the pellet re-suspended in 10 ml of 50 mM Tris–HCl (pH 7.5). The filtration and spin was repeated and the pellet resuspended in 6 ml of 50 mM Tris–HCl. This bacterial cell suspension was over-laid on 5 ml of 50% (w/v) Iodixanol-Tris–HCl solution and centrifuged at 4,000 rpm for 40 min at 10°C. Approximately 0.5 ml bacterial cell fraction was collected with a sterile glass capillary dropper and diluted with an equal volume of sterile water and centrifuged at 10,000 rpm for 1 min. The pellet was then resuspended in 350 μl sterile water and an aliquot of 20 μl immediately plated onto L-Broth agar plates prior to storing at −80°C.

### DNA Extraction and Sequencing Library Preparation

Bacterial pellets were resuspended in 200 μl TE buffer (Tris-HCl, 10 mM; EDTA, 1 mM; pH 7.0) for 30 min. The DNA was extracted with N-acetyl-N-trimethyl ammonium bromide (CTAB)/NaCl. DNA was precipitated overnight at −20°C with 70% ethanol before purification by phenol/chloroform/isoamyl 25:24:1 and chloroform/isoamyl 24:1 cleaning. The final DNA precipitate was dried and resuspended in 35 μl TE buffer and stored frozen (−20°C), after the protocol recommended by the DOE Joint Genome Institute (JGI) for isolation of genomic DNA from bacteria (http://my.jgi.doe.gov/general/protocols.html).DNA extracts were quality checked using agarose gel electrophoresis and quantified using a spectrophotometer (Nanodrop ND-1000; NanoDrop Tech. Inc). Samples containing a sufficient yield of high mass and purity DNA were selected, at approximately 50 ng of DNA template per sample. The V4 variable region of the 16S rRNA gene was amplified using primers F515 (5′-NNNNNNNNGTGTGCCAGCMGCCGCGGTAA−3′) and R806 (5′-GGACTACHVGGGTWTCTAAT−3′) (Caporaso et al., 2011), including a unique 12 bp index sequence in each of 24 reverse primers to allow samples to be multiplexed on a single sequencing run. Primers also included sequencing adapters to allow the products to be directly sequenced on Illumina platforms, and were supplied in the NEXTflex 16S V4 Amplicon Sequencing Kit (Bioo Scientific)

PCR conditions consisted of an initial 98°C for 2 min denaturation followed by 30 cycles of 98°C for 45 s, 50°C for 60 s, and 72°C for 90 s, and a final extension of 72°C for 10 min. PCR clean-up used a bead based clean-up and size selection step (Agencourt XP Ampure beads). The quality of the final products was quantified by gel electrophoresis and Qubit fluorometer (Life Technologies). The samples were diluted to 1 ng/μl and pooled in a 4 nM library and sequenced using the 250 bp paired-end protocol on an Illumina MiSeq.To prevent potentially low diversity reads causing focusing errors 10% PhiX was spiked in the pooled library.

### NGS Data Processing

Raw Illumina *fastq* files were de-multiplexed, paired, quality filtered, and analyzed using QIIME v1.8.0. (Caporaso et al., 2010). Sequences were excluded if a quality score phred < 33 was detected in 75% or more bases, if reads contained one or more ambiguous base call, or if reads were < 190 bp. Operational taxonomic units (OTUs) were defined and classified taxonomically using QIIME's uclust-based open-reference OTU-picking workflow, with thresholds of 97% pairwise identity, and 97% similarity (Edgar, 2010). Reference-based OTU picking was performed using a representative subset of the Greengenes bacterial 16S rRNA database 13_8 release (DeSantis et al., 2006; McDonald et al., 2012). Bacterial 16S rRNA gene sequences were aligned using PyNAST (Caporaso et al., 2010) against a template alignment of the Greengenes core set filtered at 97% similarity. From this alignment, chimeric sequences were identified and removed using ChimeraSlayer (Haas et al., 2011) and a phylogenetic tree was generated from the filtered alignment using FastTree v2.1.3 (Price et al., 2010). Sequences failing alignment or identified as chimeric were removed before downstream analysis. Also removed were low coverage samples with <11950 reads.

### Data Analyses

Effects of soil origin and host species on plant growth were tested using two-way ANOVAs in R version 3.2.4 (R Development Core Team, 2008). Normality and heteroscedasticity were checked graphically with quantile-quantile plots. Differences between means in ANOVAs were checked *a posteriori* with the Tukey HSD test. Host trait data distribution was slightly right-skewed so data were log-transformed to meet the assumptions of analyses.

Alpha-diversity (within-sample species richness) and beta-diversity (between-sample community dissimilarity) estimates were calculated within Phyloseq version 1.12.2 (McMurdie and Holmes, 2013) using weighted UniFrac (Lozupone and Knight, 2005) and Bray-Curtis distance between samples. OTU accumulation curves used iNEXT (Hsieh et al., 2016). Alpha-diversity estimates based on Shannon's index were presented here, this method is pragmatically applied to microbial communities as it accounts for both abundance and evenness of the species present (Hill et al., 2003; Morris et al., 2014; Kim et al., 2017), other alpha-diversity indices were explored and gave qualitatively similar results between hosts. Non-metric multi-dimensional scaling (NMDS) ordinations of community structure were computed from the resulting distance matrices and plotted in R using ggplot2 version 1.0.1 (Wickham, 2010) to visualize sample relationships as clusters.

Six factors: Soil type, Host genus, Sequencing run, Soil sub-site, Pot replicate, and Library size were plotted and tested for significant structuring effects on root associated bacterial community diversity. ANOSIM and ADONIS functions were implemented in R via VEGAN version 2.0-10 (Oksanen et al., 2008) and permutational MANOVA with 999 permutations used to test significant differences between sample groups based distance matrices. Differential expression analysis implemented in R used DeSeq2 version 1.8.0 (Love et al., 2014) to model (bacterial diversity _~_ soil^*^host^*^replicate) and test, by multiple contrasts, which taxa differed significantly between sample groups. Data can be accessed from the NCBI (SDR) under the BioProject ID: PRJNA517105, accessions SAMN10819603 - SAMN10819674. Details of analysis can be found (Supplementary Material 2).

## Results

### Soil Treatments: Composition of the Different Soils

The abiotic characterization revealed higher nutrient and moisture content in woodland than grassland soils, and higher variance in woodland soils (Table 1). Mean soil moisture [*F*_(1, 4)_ = 6.7, *p* = 0.06] and nitrogen content [*F*_(1, 4)_ = 4.7, *p* = 0.09] differed significantly between soil types. Nitrogen assays showed consistently higher N content in woodland soils, irrespective of method (*r*^2^ = 0.94).

**Table 1:** Soil nutrient and moisture content (means ± S.E.M).

**Soil type**	**Total.N^†^**	**Total.P^†^**	**Ex.Am^†^**	**Ex.Nit^†^**	**Ex.P^†^**
Grassland	3,385 ± 374	380 ± 20	10.1 ± 0.51	0.1 ± 0.06	1.8 ± 0.17
Woodland	8,028 ± 2,268	570 ± 122	33.9 ± 15.7	1.1 ± 0.78	8.8 ± 6.1
**Soil type**	**N^‡^**	**C^‡^**	**C:N^‡^**	**Moisture Content**^Θ^	
Grassland	0.27 ± 0.12	0.2 ± 0.01	13.5 ± 0.25	0.075 ± 0.002	
Woodland	11.7 ± 4.5	0.7 ± 0.2	15.7 ± 1.26	0.727 ± 0.252

† values in μg/g dry weight;

‡ values in % total mass;

Θ *values as fraction dry weight*.

### Host Treatments: Variation in Host Growth

**Figure 1:**
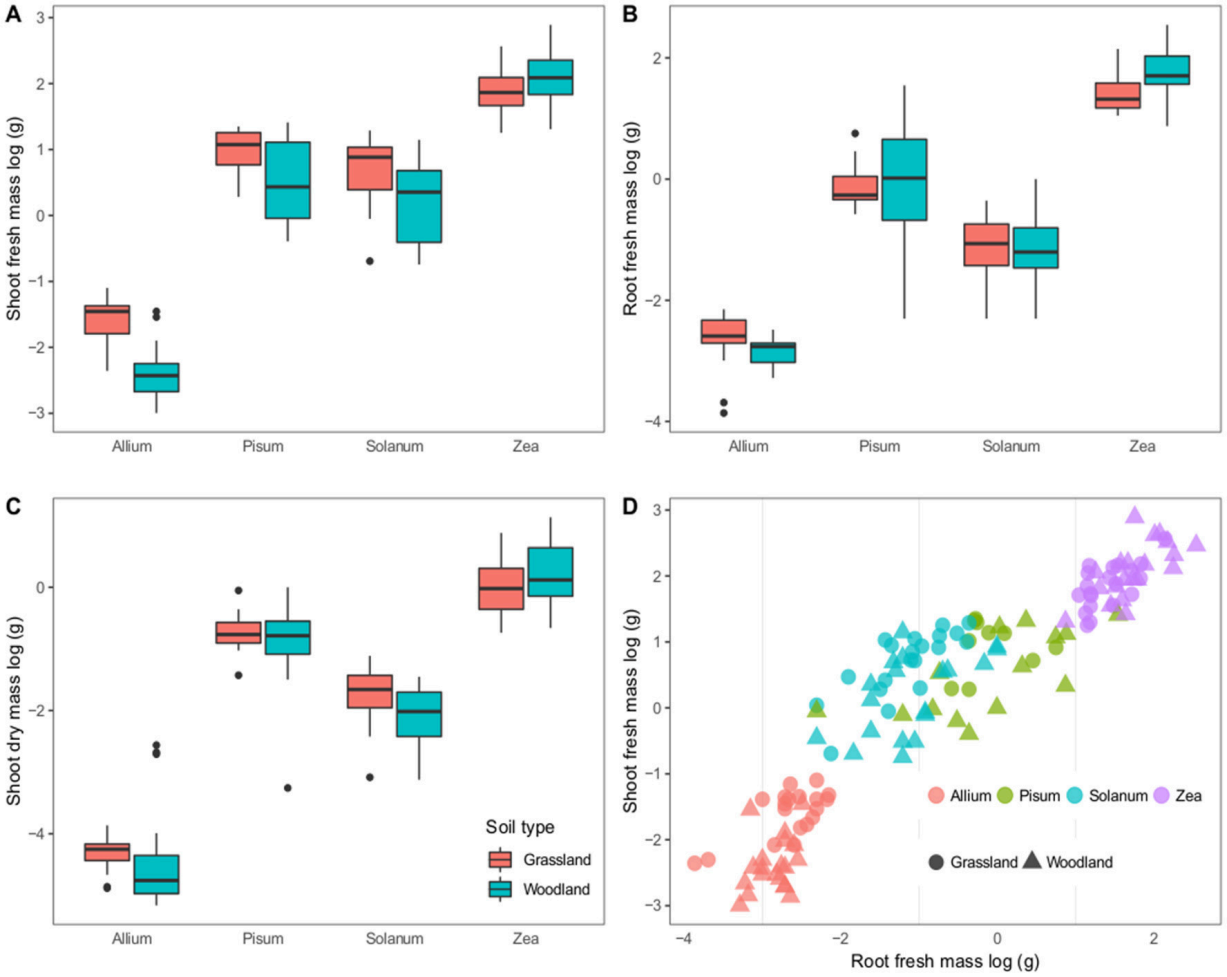
Variation in plant growth between soil types. The first three panels show boxplots of log–transformed masses; **(A)** Shoot fresh mass, **(B)** Root fresh mass, and **(C)** Shoot dry mass. Panel **(D)** shows a scatterplot of the root vs. shoot masses. Colors correspond to host genera and shapes to soil types. The boxplots show a mean as a thick horizontal bar, the body of the box is the lower and upper quartiles (25 and 75%), the whiskers show the 5–95% range and outliers are black points.

The four hosts have distinct growth patterns that showed little variability between the two soil treatments (Figure 1). The most striking difference was that all Pisum plants showed root nodules when grown in grassland soil (*n* = 46 plants) but never in woodland soil (*n* = 56). Across all host plants fresh shoot mass varied most between soils: *Allium and Solanum* plants increased fresh shoot mass in grassland soils [*F*_(2, 37)_ = 38.8, *p* < 0.0001] and [*F*_(2, 37)_ = 7.3, *p* = 0.01, Figure 1A] respectively. Root mass also showed limited variability between soils, although *Zea* plants had increased root mass on woodland soils [*F*_(2, 37)_ = 9.1, *p* = 0.004, Figure 1B]. *Solanum* were the only plants to show a response to soil type in terms of mean shoot dry mass, being higher on grassland soil [*F*_(2, 37)_ = 5.14, *p* < 0.05, Figure 1C). Root shoot ratios were also relatively consistent for distinct crop plants, regardless of soil type, except for *Solanum* where soil type was an influence [*F*_(2, 37)_ = 9.1, *p* < 0.005, Figure 1D].

On the whole fewer plants established in the woodland soil compared to the grassland soil (Supplementary Table 1). This difference is significant for Solanum and Zea plants that showed a 16 and 12.6% decrease in establishment (p < 0.001 and p = 0.025 respectively).

### Root Associated Bacterial Communities

**Figure 2:**
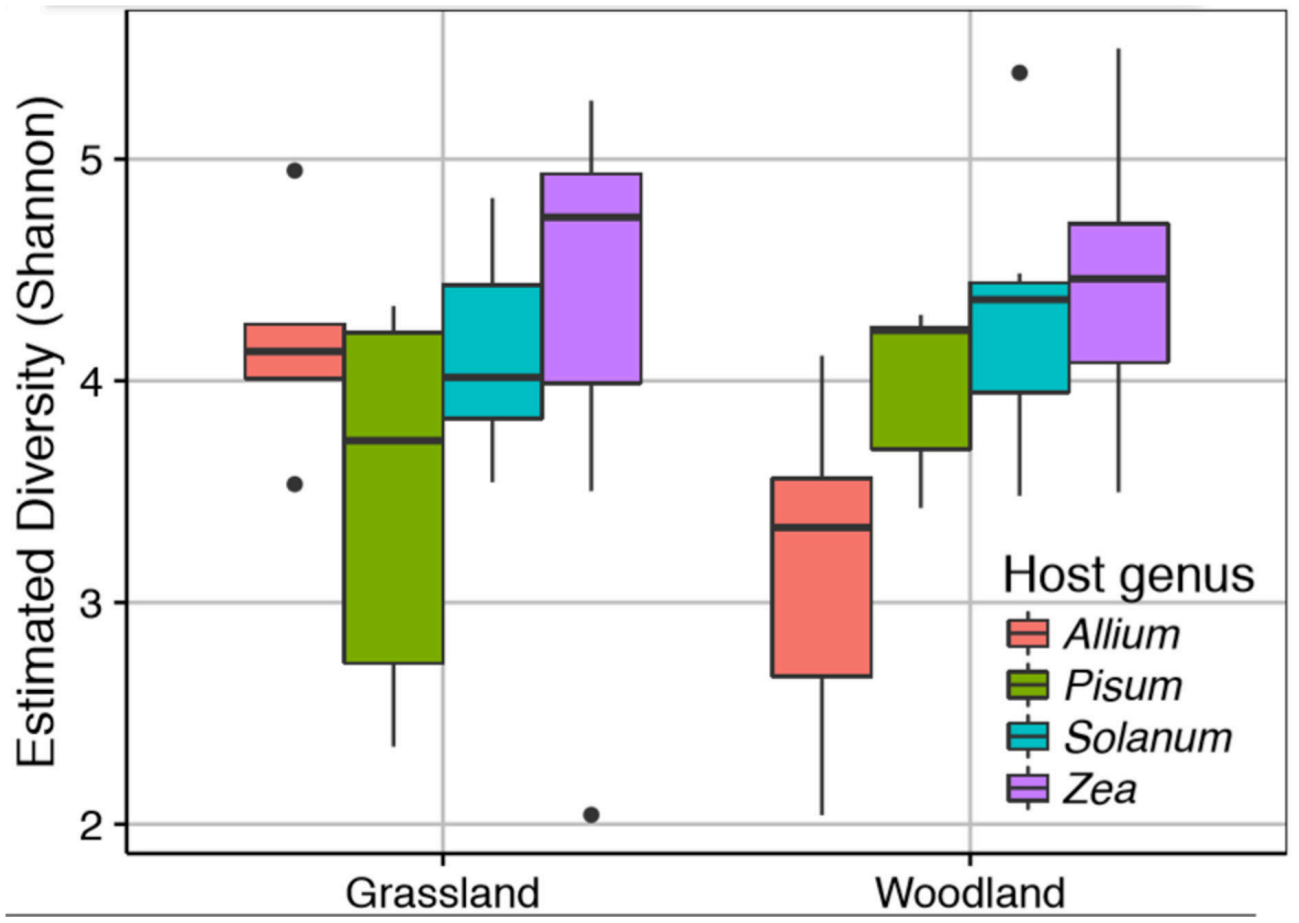
Variation in root associated bacterial community alpha diversity. The observed diversity is shown at a normalized library size of 10k reads. Colors indicate host plants, while Grassland and Woodland indicate the two soil treaments.

We sequenced the V4 region of the 16S rRNA gene of the root associated microbial communities from 72 plant rootstocks. Of these five samples fell below the minimum sequencing depth cut off. Of the 67 remaining samples 32 were from plants grown in a grassland soil comprising *Allium n* = 5, *Pisum n* = 6, *Solanum n* = 10, and *Zea n* = 11. From the woodland soil we sequenced 35 plants comprising *Allium n* = 7, *Pisum n* = 7, *Solanum n* = 9, and *Zea n* = 12. Detailed information on sample quality control and normalization are provided Data Sheet 1. Neither soil type nor its interaction with plant species had a strong impact on alpha diversity (Figure 2). There was, however, significant variation in Shannon diversity associated with host genus [ANOVA: *F*_(3, 63)_ = 8.18, *p* ≤ 0.0001). A *post-hoc* Tukey test showed mean observed OTUs in *Zea* root bacterial communities (1003 ± S.E. 52) was significantly higher than in *Solanum* (828 ±S.E. 44, *p* < 0.05), *Pisum* (706 ±S.E. 41, *p* < 0.001), and *Allium* (690 ±S.E. 63, *p* < 0.001) but that *Allium, Pisum*, and *Solanum* communities had similar species richness (*p* > 0.3).

**Figure 3:**
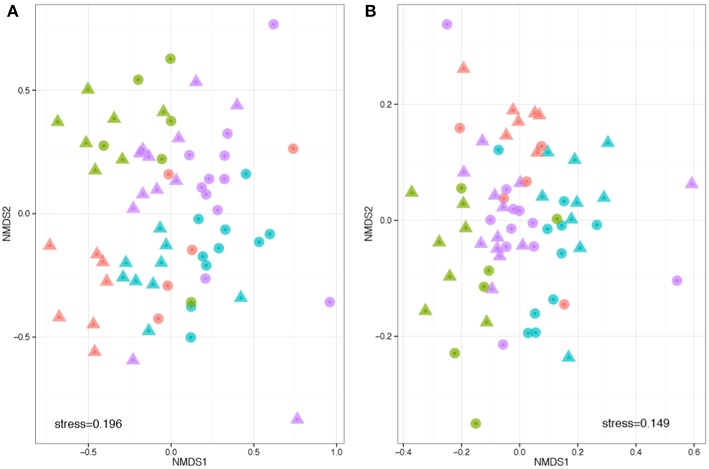
Phylogenetically naïve and phylogenetically informed analyses of the impact of plant and soil on community structure. Compositions were compared based on a rarefied OTU table, with OTUs defined at 97% sequence similarity threshold. Non-metric multidimensional scaling analysis was performed based on pair-wise between-sample, normalized Bray–Curtis **(A)** and weighted UniFrac distances **(B)**. The stress value indicates the degree of fit between the original distances in the matrix and the reproduced distances within the ordination plot. Each point represents a single sample. Note that the hosts (colors) are more obviously clustered than the soils (shapes). Legend key as in Figure 1D.

Beta diversity describes the compositional heterogeneity between samples. Difference in total bacterial species composition showed a robust effect of plant species, but also a significant effect of soil. Experimental replicate had no significant effect on community structure. The phylogenetically naïve analysis of beta diversity (Bray Curtis dissimilarity) showed significant clustering and differences in the community structure between soils, plant species and a significant plant soil interaction (ANOSIM: *R*_plant_ = 0.32, *F* = 9.3, *p* = 0.001; *R*_soil_ = 0.32, *F* = 5.8, *p* = 0.001; *R*_plant:soil_ = 0.48, *F* = 1.8, *p* = 0.001, Figure 3). However, when taking into account pairwise phylogenetic distance between OTUs in the community structure analysis (NMDS with Weighted UniFrac), plant species had much stronger effect than soil and there was a reduced interaction between the two (ANOSIM: *R*_plant_ = 0.39, *F* = 8.5, *p* = 0.001; *R*_soil_ = 0.07, *F* = 1.8, *p* = 0.001; *R*_plant:soil_ = 0.417, *F* = 1.5, *p* = 0.033, respectively, Figure 3). These analyses reveal that specific bacteria tend to be associated with different host species. The effects of soil are more complex: within crop species plants associate with different OTUs in different soil types, but these OTUs tend to be drawn from phylogenetically similar groups.

### Relative Abundance of Taxa Across Soils and Hosts

A total of 8,210 unique OTUs clustered at 97% similarity were analyzed within the rarefied data set. A taxonomic summary, showing the average abundance of bacterial families in samples organized by soil and host, illustrates the compositional differences between root associated bacterial communities (Figure S5). Although there is between sample variance, the general patterns are clear. Six dominant phyla overall account for 99.4% of the OTUs found: Proteobacteria 83.5%, Actinobacteria 7%, Verrucomicrobia 3.6%, Planctomycetes 2.8%, Bacteroidetes 1.3%, Firmicutes 1.2%. Phylum level patterns emerged in response to soil and crop species. *Pisum* plants in both soils had consistently the highest relative abundance of Proteobacteria in grassland (mean 94%, range 82–99%) and woodland, (mean 96% range 94–98%) than other host soil combinations (mean 80%, range 6–99%). Proteobacteria had a decreased average relative abundance in woodland grown *Solanum* (mean 68%, range 51–80%) compared to other host soil combinations (mean 86%, range 6–99%). Actinobacteria also showed a general trend toward increased relative abundance in *Allium* and *Solanum* plants as opposed to *Pisum* or *Zea* (12 and 2%). The greatest variation in relative abundance within sample group originated from two *Zea* samples, these samples had notably decreased Proteobacteria (typically 9%) but increased Verrucomicrobia (typically 73%) relative abundance, respectively.

**Figure 4:**
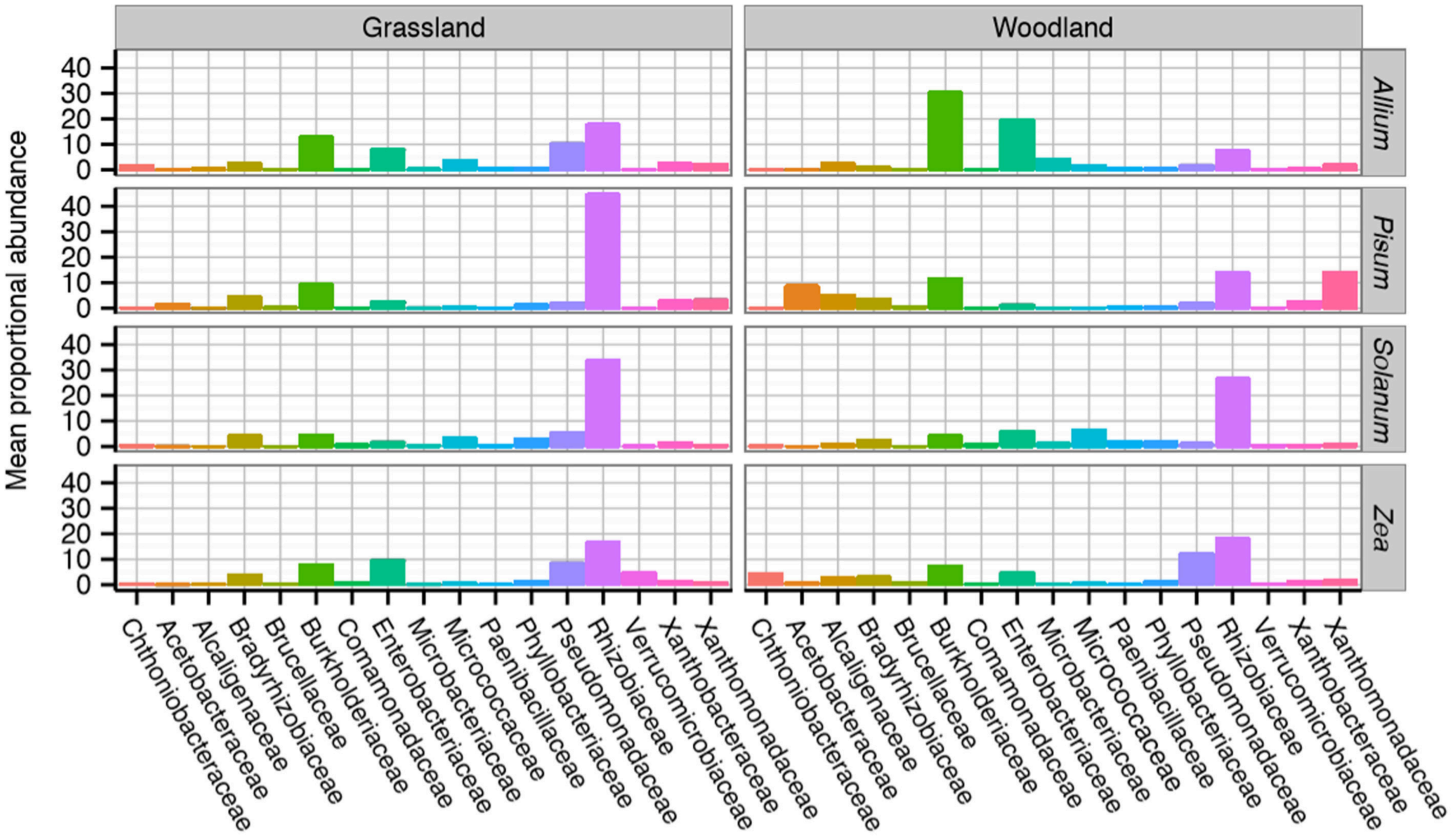
Average taxonomic composition of bacterial communities associated with plant roots. Compositional differences were observed between host genus and soil type across taxonomic ranks. Data shown here are average composition by host and soil at the Family rank. Multiple replicates are summarized with the height of each bar representing the mean proportional abundance of those taxa in the normalized samples. For a more detailed breakdown of compositional changes by rank see Figure S4.

Family level compositional shifts are summarized in Figure 4. Within Proteobacteria the most common families were Rhizobiaceae (average = 44%, range 5–83%), and Pseudomonadaceae (average = 32%, range = 2–81%) with substantial variation at the family level between crop species and soil combinations. A close examination of OTU relative abundances by families shows patterns of repeated differences in community composition between plant taxa. In *Allium*, particularly those cultured in woodland soil, Burkholderiaceae, and Enterobacteriacae are prevalent, making up 33%, and 22% of the communities. In *Pisum, Zea* and *Solanum*, Burkholderiaceae and Enterobacteriacae are far less abundant, making up 9 and 5% of the community overall. *Pisum* plants grown in woodland soil are peculiar in their enrichment of OTUs from the Acetobacteraceae (average = 17%, range = 9–41%) and Xanthomonadaceae (average = 21%, range = 2–59%), while these families make up only 1 and 4%, respectively, of communities associated with other plants. Pseudomonadaceae present increased relative abundances in *Zea* (average = 14% range = 1–48%) compared to all others (average = 4% range = 0–37%). Otherwise there are clear soil plant interactions: Pseudomonadaceae are much more abundant in both grassland grown *Allium* (13%) and *Solanum* (7%) than their woodland counterparts (2% for both; Figure 4).

### Differentially Abundant OTUs

**Figure 5:**
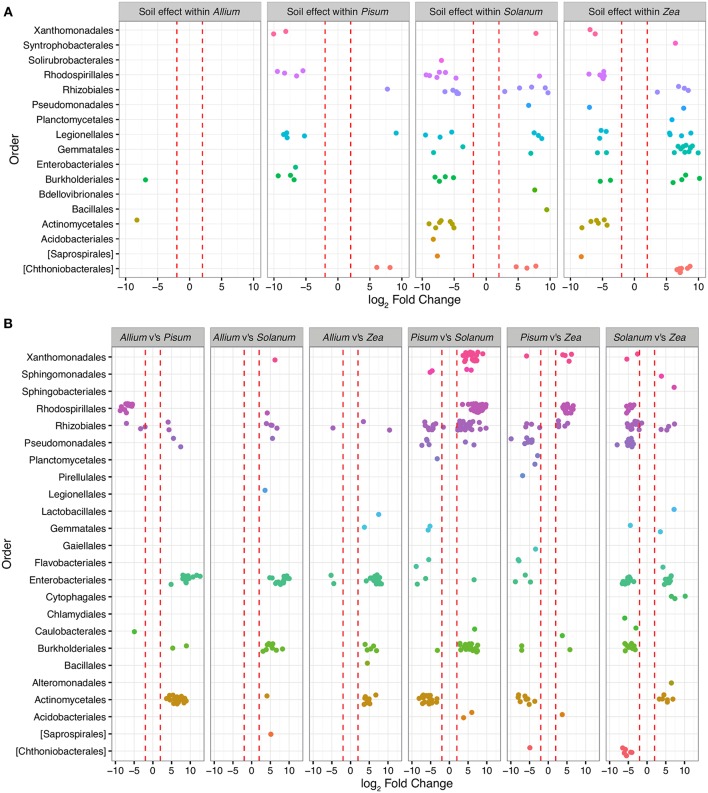
OTUs colored by Order and organized in panels summarizing the main contrasts. Each point represents an OTU that was significantly (*P*-value adjusted for multiple testing *p* < 0.0001) differentially represented between treatment groups. Red dashed lines mark the threshold of change in normalized abundance required to constitute a differentially abundant OTU, i.e., 2 ± log-2-fold change. **(A)** shows within host shifts in OTUs between soil types grassland compared to woodland. Here each point represents an OTU that was significantly differentially represented between soil types within a host genus. **(B)** shows taxa driving the differences between hosts when soil type is ignored. Each contrast is made for the host genus on the LHS compared to the RHS in each sub plot.

To determine those taxa strongly enriched or depleted by a treatment, we applied an analysis based on models of differential expression (Paulson et al., 2013). Taxa associations were considered significant if normalized abundances were log-2-fold increased or decreased between the base mean of any given contrast. Mean log-2-fold changes in abundance by family and contrast are presented in Table ST2. Only 479 OTUs (approximately 6% of total) were significantly differentially abundant (adjusted *p* < 0.00001) in pair-wise comparisons across soils or plants, and of these Proteobacteria account for 71% differentially abundant taxa, Planctomycetes 11%, Actinobacteria 9%, Verrucomicrobia 5%, and Bacteriodetes 2%. Between soil types, 156 OTUs are differentially abundant, whereas between plants 231 OTUs differ, with 59 interacting with soil and host. OTUs over-represented in grassland relative to woodland soil sit within the Planctomycetes (*n* = 40; average LFC = 4.8), Verrucomicrobia (*n* = 17, average LFC = 4.9), while under-represented taxa are from Acidobacteria (*n* = 4; average LFC = −4), Actinobacteria (*n* = 16; average LFC = −4.3), β-Proteobacteria (*n* = 29; average LFC = −4.5; Figure 5). Soil did not have a similar effect on all crop species. In particular, very few OTUs varied significantly between soils for *Allium. Pisum* communities were enriched in four sub-phyla in woodland soils but for relatively few OTUs (Figure 5). Soil also had an important effect on *Solanum* and *Zea* communities, both in enrichment of particular clades or in changing the representation of OTUs within sub-phyla.

Patterns of association vary substantially depending on taxonomic level. Exploration of family level associations showed frequent plant bacteria associations in γ-Proteobacteria (Enterobacteriaceae, Pseudomonadaceae, Xanthomonadaceae, Figure 5). Notably, Moraxellaceae is relatively rare in *Pisum* compared to other hosts (Figure 5). However, occasionally we see evidence for plants consistently associating with particular groups of OTUs within family. *Zea* and *Solanum* for example, associate with distinct sets of OTUs within the Enterobacteriacae, while *Pisum, Solanum*, and *Zea* associate with distinct OTUs within the Rhizobiaceae (Figure 5B).

## Discussion

Distinct bacterial communities are typically associated with roots of different agricultural crop species in the field (Wieland et al., 2001; Marschner et al., 2004; Haichar et al., 2008). However, crop species effects are difficult to identify because distinct communities may be the consequence of differences in local site or soils. In other words, site and plant effects are commonly confounded in studies of crop microbiomes. Here, we have shown that root bacterial communities vary significantly between hosts, even after controlling for soil type and growth conditions. A handful of studies have tried to separate the relative importance of soil type and host plant on bacterial rhizosphere communities (Bonito et al., 2014; Ofek-Lalzar et al., 2014; Pii et al., 2016). However, these studies were often done with low taxonomic resolution. For example, analyzing community effects at the phylogenetic level of Order or Class often masks taxonomically important variation (Beckers et al., 2016). In other cases, low sample size (*n* = 2–10) or insufficient number of reads per sample has meant that complex experiments were not fully characterized. This study uses in depth sequencing of experimental communities and confirms that both plant and soil are important, but that the interaction between the two can vary with host species.

Host specificity in root associated microbial communities is thought to derive from the existence of plant-microbe co-adaptation, involving a shared evolutionary history of interactions between plants and microbiota (Knack et al., 2015). In fungi, for example, host specificity is ancient and well-known (Becker and Marin, 2009; Morris et al., 2015). In plants, genetic variation for regulating bacterial associations in the rhizosphere is becoming better characterized (Schlaeppi et al., 2014; Panke-Buisse et al., 2015; Wintermans et al., 2016) and can have marked consequences for fitness; for instance via bacterially-mediated increases in shoot fresh weight and changed root architecture (Callaway et al., 2007; van der Heijden et al., 2008; Haney et al., 2015). Plant communities are thought to play a substantial role in both soil formation and the emergence of distinct microbial communities in soil (Bever et al., 2012; Schweitzer et al., 2014; Terhorst et al., 2014). But in order to make the switch to more intimate association with roots or the rhizosphere, a substantial suite of metabolic, secondary metabolite and secretory genes are required (Roumet et al., 2016; Roux and Bergelson, 2016). These are likely to be costly, and therefore might impose constraints on host range (Bakker et al., 2013). Conversely, while plants can accrue benefits from their microbiota, they are also subject to attack from host-specific pathogens, a selection pressure that is likely to drive the ability to discriminate or control host associated assemblages (Kinkel et al., 2011; Oldroyd, 2013; Wu et al., 2015). High levels of discrimination, however, are likely to be constrained by the pool of available partners and balanced by the cost of discrimination or elevated immunity (Steidinger and Bever, 2014).

The variation in the functional role provided by rhizobacteria and the balancing constraints of costly discrimination are likely to lead to between species variation in the way plants structure microbial communities. Bulgarelli et al. (2013) propose a two-step selection model of community assembly; initially host driven root exudates drive metabolic selection of microbes in the rhizosphere, then host genotype dependent fine tuning controls selection of suitable endophytes at the root surface. This model reflects the observed shifts in abundance and attrition in diversity within these niches (Edwards et al., 2015). In this model the magnitude of selection in the rhizosphere is likely to vary in different plant species and as a function of the host genotype. This assumes that hosts may be more or less selective in acquiring rhizobacteria and implies that rules of community assembly may vary between plant species.

In this study, the way in which rhizosphere communities either do or do not vary with soil types suggests that some hosts may indeed be more discriminating. For example, *Allium* has a relatively invariant community make-up irrespective of soil, only two OTUs (from a total 692 OTUs) are significantly differentially abundant in these different environments. *Pisum* also possessed a relatively fixed community structure with 18 OTUs (out of 706) varying in relative abundance between soils (Figure 5), although when variation did occur OTUs were enriched from distinct phylogenetic groups (Figure 4). In contrast, *Solanum* and *Zea* have community structures that vary substantially across soil types (Figure 5) but OTUs were often drawn from particular orders, especially for *Zea*. As a result differences are not so apparent in comparisons at high level taxonomic rankings. These patterns suggest that community assembly itself is plastic across plant species, varying from relatively invariant to dynamic with respect to soil type.

In addition, competitive/cooperative interactions within the community or priority effects may shape community assembly. *Zea* communities, for instance, have the most dispersed and diverse communities (Figures 3, 5) relative to other plant species. While the most common community type is dominated by alpha Proteobacteria, the next most common community has a distinct but also consistent make-up (Verrucomicrobia dominated) regardless of the soil type in which the hosts grew. Speculatively, community assembly in *Zea* may take the forms of multiple alternative stable states (van der Heijden and Hartmann, 2016). Experimental results in simple systems suggest that dynamic community assembly scenarios such as alternative stable states do occur (Tkacz et al., 2015). These alternative stable states may be the result of priority effects, such as community assembly, member order effects or competition structuring effects (Bever et al., 2010; Agler et al., 2016). Potentially, keystone taxa such as Actinobacteria can limit diversity in rhizosphere communities as they do in soil (Agler et al., 2016; Jung et al., 2016). Also, competition within the root system can lead to spatial variation in bacterial community diversity, where for instance community composition responds to various co-occurring fungi, differing from root tip to root tip (Marupakula et al., 2016).

It is well-documented that members of the Alliaceae have distinct anti-microbial profiles including flavonoids and glycosides with broad antimicrobial activity (Deberdt et al., 2012; Arnault et al., 2013). We might, therefore, expect a strong host plant signature in their root bacterial communities. Our results support this: *Allium* had a distinct composition in terms of Actinobacteriales, Entobacteriales, and Burkholderiales. *Allium* roots were enriched in Actinobacteria including Microbacteriaceae, Micrococcaceae, and Frankiaceae. Currently, only a single study of bacteria associated with *Allium* roots has been conducted using high-throughput sequencing (Gardner et al., 2011), and also found significantly more Actinobacteria in *Allium* compared to the rhizospheres of other hosts grown in the same soil or bulk soil. Furthermore, *Allium* bacterial communities are peculiar in their elevated abundance of a single OTU from the Bradyrhizobiaceae (*Balneimonas*), which had increased relative abundance particularly in woodland soil. The production of extracellular polysaccharides (EPS) is an important factor in the development and survival of *Balneimonas* species, and has been attributed to their function in bacterial soil crusts (Elliott et al., 2014). Alliaceae are notable for their mucilaginous root; the production of mucilage or EPS reduces friction as their roots ramify through soil. It is plausible that an association with copious biofilm producing bacteria such as *Balneimonas* contributes to this phenotype (Augimeri et al., 2015).

In animals the emerging model of microbial community assembly in the gut is a nested hierarchy of factors complicated by interactions such as diet (Voreades et al., 2014; Wang et al., 2014) and metabolic and behavioral cycle of the host (Thaiss et al., 2014). By contrast the relative importance of factors shaping rhizosphere community assembly in roots is less clear. Not only are root associated microbial communities continuous with the environment and generally an order of magnitude more diverse than gut communities, but different assembly rules appear to be at play for different taxa. For instance the role of local conditions and spatial scale appears to be paramount for fungi (Maherali and Klironomos, 2012; Lagunas et al., 2015; Rúa et al., 2016) whereas host genotype and compartment seem to be more important factors in rhizosphere bacteria (Hacquard, 2016). Community assembly rules certainly varied in this study; ranging from both relatively constant to highly variable with respect to soil. As yet we cannot determine if variable community composition is due to a lack of discrimination, or whether different plants select different microbes in different habitats to help shape environment-dependent fitness, or indeed if there is variable colonization driven by bacterial responses. (Rodriguez et al., 2008; Rolli et al., 2015;Hiruma et al., 2016).

There is the suggestion that domestication and soil management have limited the evolutionary potential of microbial interactions in crop associated microbes (White et al., 2013; Perez-Jaramillo et al., 2016). Improved application of bacteria in agriculture could benefit from better understanding rhizosphere community assembly patterns. For instance, if crop species such as onion are highly discriminating, it would make sense to select PGPR that are members of a known crop associated community. Conversely, attempts to alter community composition may be more successful in species such as maize, which has a more variable community, with the caveat that we do not yet understand if variable communities imply less discriminating assembly. These results predict that blanket application of generic PGPR may be at best a naive strategy, as their potential effect on microbiome composition and plant performance may be limited by restrictions on host range, establishment, or invasion. A challenge for the future understanding of the ecology of PGPR and rhizobacterial communities is to identify when communities in agro-ecosystems limit plant yields and to use the available molecular tools to explore strategies to manipulate communities effectively.

## Data Availability

The datasets generated for this study can be found in NCBI SDA, BioProject ID: PRJNA517105; accessions SAMN10819603 - SAMN10819674.

## Author Contributions

The study was designed by AM and BR. Experimental work was undertaken by AM with NGS assistance from HH and soil analysis from SP. Data analysis and draft manuscript by AM. All authors contributed to the final manuscript.

### Conflict of Interest Statement

The authors declare that the research was conducted in the absence of any commercial or financial relationships that could be construed as a potential conflict of interest.
