# Supplementary material for: Rhizobacterial Community Assembly Patterns Vary Between Crop Species
Source: Front Microbiol. 2019 Apr 4;10:581. doi: 10.3389/fmicb.2019.00581 (PMC6458290; doi:10.3389/fmicb.2019.00581)
Supplement: Supplementary file 1 [file Data_Sheet_1.docx]

**Supplementary Materials**

*Site Description*.

Soil types are free-draining, low base status (pH is 5) sandy soil. Soil is nitrogen, phosphorus and base deficient. Soil is mapped as Bagshot sands with some plateau gravels.

The site history is described in detail in the Flora of Berkshire; its underlying geology is thought to be similar between the two soil types in this study, as they share parent material originating from the local Bagshot sands. They are pedomorphologically distinct being overlaid both historically (since 1650’s) and today by different vegetation types comprising a woodland and grassland. The sites were chosen for and described after their extant vegetation cover types described briefly below.

##
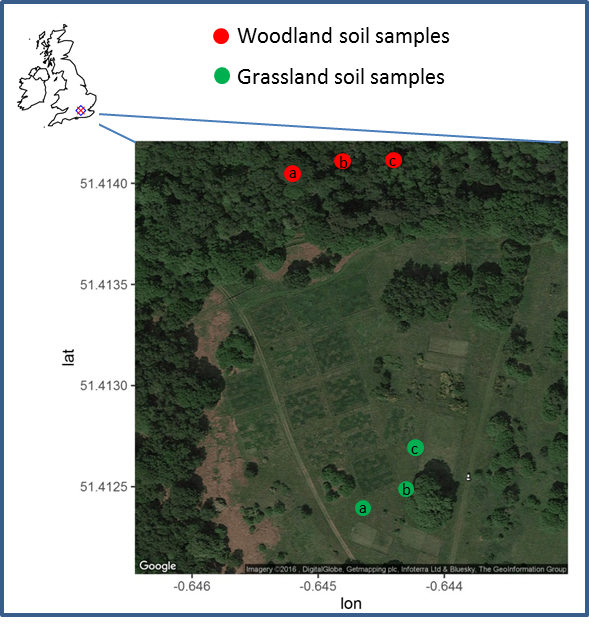


Figure S1: Site map, soils were collected from a Woodland (Red) and Grassland (Green habitat within Silwood Park, near Ascot in South East UK.

Nash’s Field an acid mesotrophic grassland NVC:MG5 (Rodwell, 1992). Vegetation cover is dominated by *Cynosurus cristatus & Centaurea nigra*, the sub community is defined by the presence of *Danthonia decumbens*.


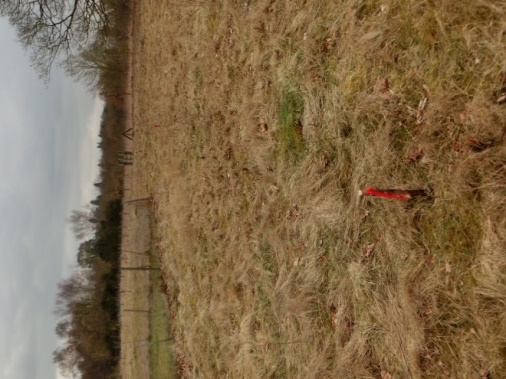
Figure S2: Nash’s Field in March 2013 showing the dead thatch of previous season grass growth. Typical (rank sward) vegetation cover in early spring. Red marker in foreground shows position of subsite a.

Three 40L soil samples were recovered from trenches dug along the southern border of an existing long-term field experiment described in (Edwards and Crawley, 1999, Allan and Crawley, 2011).


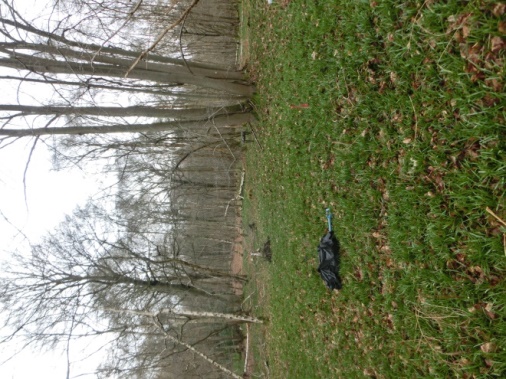
Nash’s Copse NVC:W10 is a *Quercus robur* - *Pteridium aquilinum – Rubus fruticosus* woodland with a canopy dominated by *Betula pendula* and occasional *Fagus sylvatica,* *Hyacinthoides non-scripta* is a locally dominant member of the ground layer at the soil sampling sub sites.

Figure S3: Nash’s Copse, March 2013 showing typical early spring flush of *Hyacinthoides non-scripta* and *Betula pendula* flowering. Three 40L soil samples were recovered from trenches dug following a contour 15m from the Silburn stream flowing from Silwood lake.

**Soil characterisation methods**

Fresh soil was taken from each subsite sites with an auger to a depth of 10 cm, using five soil cores per sampling site (total cores n=30). Fresh soil passed through a sieve (ca. 2mm) and weighed. Available free nitrogen (nitrate/nitrite and ammonia) was extracted by mixing 20g of fresh, sieved soil with 75ml of 1M KCl. Available phosphate was extracted by mixing 10 g of fresh, sieved soil with 150ml Truog’s solution (995ml H_2_0 3g (NH_4_)_2_SO_4_ 5 ml of 0.05M H_2_SO_4_), both extracts were shaken at 150rpm for 60 & 30 minutes respectively then stored over night at 4 °C. Soil moisture content was measured by weighing ca. 10g of each fresh soil in to paper bags, drying at 40 °C and re-weighing 5 days later. Soil moisture was calculated as (wet soil weight – dry soil weight) / dry soil weight) * 100 = % soil moisture. Total N & P were analysed with acid digests. 0.25 g of dried soil with ca. 1 g Se and 3ml of 98% H_2_SO_4_ was heated to 250 °C for 30 minutes and then to 400 °C for two hours. 15ml H_2_0 was added and the sample mixed, filtered and made up to 25ml with H_2_0. The extracts and digests were analysed with a SKALAR San++, Continuous Flow Analyzer (Skalar Analytical, Breda, The Netherlands). Additionaly C:N was measured at Forestry Research (Alice Holt, Farnham UK) using the dynamic Flash Combustion technique in a ThermoQuest EA1112 Flash Carbon/Nitrogen analyzer (ThermoFisherScientific, London, UK).

**Mesocosm set up**

Soils were mixed and screened within subsite by hand. A large plastic mortar mixing board 1.2m^2^ was used, this was washed with excess water, cleaned with 1% Distel rinsed and dried between handling independent soil subsite batches. Plastic bags were used as disposable overalls, facemasks and gloves were used to minimise contamination. This process homogenised the soils and removed detritus both solid objects greater than 2cm^3^ and vegetation excepting the finer root mass. Approximately 5L of soil was decanted in to the 7.5 L pots. Between 7-8 pots were filled per subsite to a total of 42 pots, 21 per soil type.

**Seed sterilisation validation.**

Immediately prior to sowing each batch of surface sterilised seeds was sampled and both seeds and water were plated in a hood on plant growth agar supplemented with 0.2%LB. Later that morning the same seed batches were sown directly in to pots in the glasshouse. The plates were incubated in the dark at 24°C for two weeks and inspected for signs of culturable contamination.


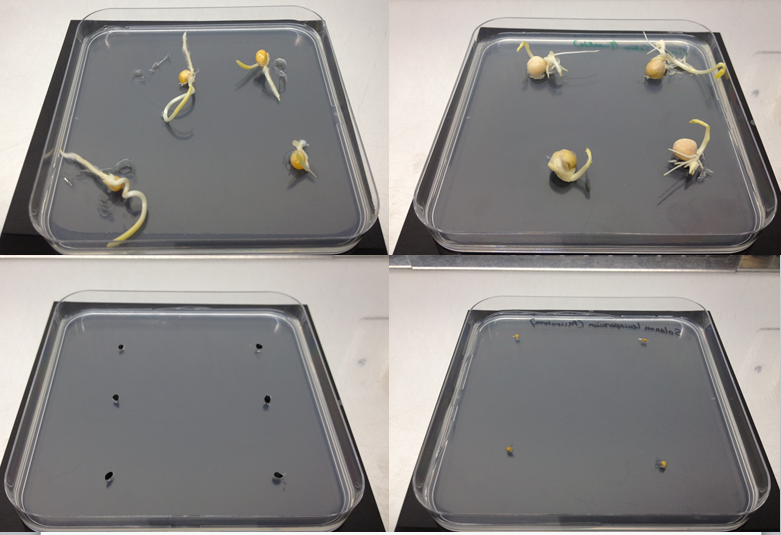


Figure S4 shows typical seed sterilisation plates with relatively uniform germination within species but, in spite of stratification, variable between species germination with larger seeded *Zea* and *pisum* relatively advanced in development compared to smaller seeded *allium* and *solanum*. After 2 weeks of germination no contaminants were cultured.

**Bacterial cell enrichment method**

Bacterial sampling methods from whole rootstocks followed established protocols (Ikeda et al 2009, Ikeda et al 2010), with some modifications. Excess soil was removed by shaking the root stock and disentangling roots on the bench prior to washing the rootstock thoroughly by agitating in an excess volume of sterile H_2_0. Whole rootstocks were then weighed and homogenised in 120ml of pre-chilled bacterial cell enrichment (BCE) buffer (1L H_2_0, 50 ml 1M Tris, 10ml Triton X100, 2 ml β-mercaptoethanol) in a blender. Samples were blended at high speed for three minutes. The homogenate was then filtered through a layer of sterilized Miracloth and centrifuged at 1,800 rpm for 5 min at 10°C to remove bulk plant debris. The supernatant was centrifuged at 4,000 rpm for 20 min at 10°C and pellet re-suspended in 5ml of BCE buffer. Samples were filtered in to one 15 ml falcon tube through 4 layers of sterilized Kimwipe. Filtrates were then centrifuged at 4,000 rpm for 10 min at 10°C. The supernatant was then discarded again and the pellet re-suspended in 10 ml of 50 mM Tris–HCl (pH7.5). The filtration and spin was repeated once and the final pellet resuspended in 6 ml of 50 mM Tris–HCl (pH7.5). This bacterial cell suspension was then over-layed on 5 ml of 50% (w/v) iodixanol solution and centrifuged at 4,000 rpm for 40 min at 10°C. The bacterial cell fraction, a whitish band located at the interface of the upper and lower phases was collected with a sterile glass capillary dropper. This enriched bacterial suspension (approximately 0.5 ml) was then diluted with an equal volume of sterile water and centrifuged at 10,000 rpm for 1 min at 10°C. The pellet was then resuspended in 350µl sterile water and an aliquot of 20µl immediately plated onto L-Broth agar plates prior to storing at -80°C.


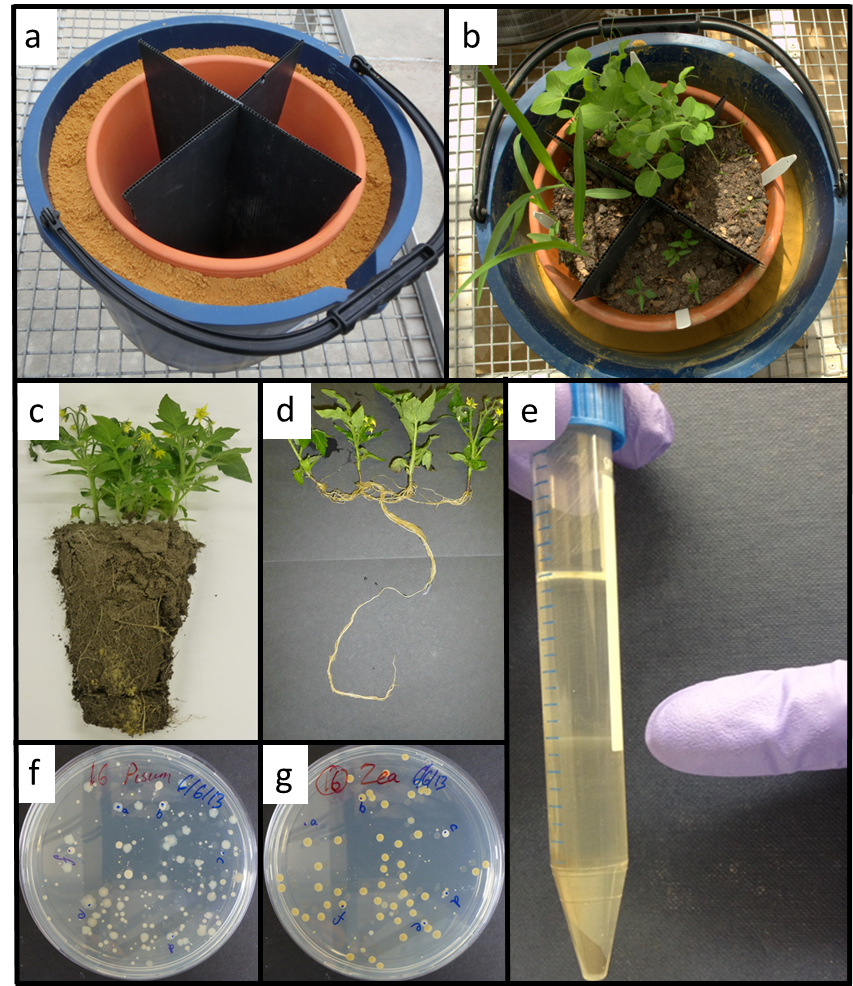


Figure S5: **a**, 13L bucket with 7.5L terracotta pot imbedded in sand and divided in to quarters by plastic dividers Correx (Cricklade, Wiltshire, UK); **b**, showing four hosts growth at approximately one month post sowing; **c**, quarter pot with divided out *Solanum* replicates; **d**, the same *Solanum* replicate after shaking and washing away soil. **e**, A density gradient centrifugation showing cloudy white band containing bacteria. Panels **f** & **g**, show *Pisum* and *Zea* replicates plated on L-broth agar.


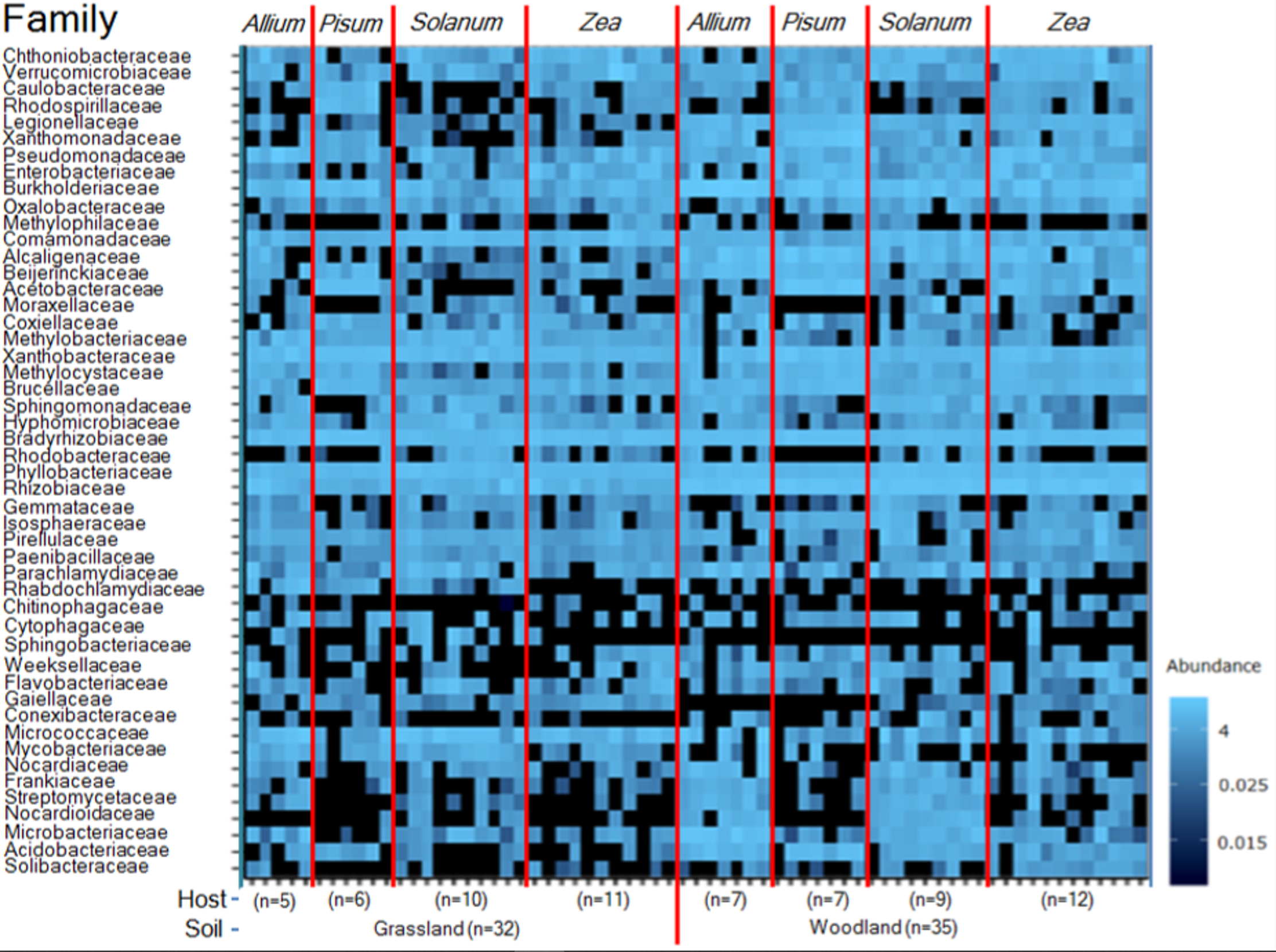


Figure S6. The sample level variation in relative abundance of total OTUs in each bacterial family. Families (rows) by samples (columns) with cell colour recording the mean normalised abundance. Light blue indicates high abundance while light blue indicates high abundance of taxa after variance stabilising transformation.

**Supplementary next generation sequencing methods.**

*Pre-sequencing normalisation*

In addition to normalising the template DNA available in 16s PCR’s the Barcoded amplicons were size checked via gel electrophoresis and quantified with a Qubit fluorometer prior to sequencing. The yields and dilutions made for 4nM library prep are included in SM1 in the file NEXTflex_AM_library_preps.xls

*Post-sequencing normalisation*

Four data pre-processing steps were used to clean and simplify the data before analysis and visualisation. Firstly a minimally pre-processed Phyloseq object lost 5 out of the 72 samples in which total sequencing depth fell below 11950 reads per sample; in addition non-bacterial OTUs were removed. This data was used in Alpha diversity, DESeq, ANOSIM and ADONIS analysis, as well as in visualisation via NMDS clustering ordinations.

Secondly, exploratory plots used sample counts transformed to relative abundance by rarefying to an even sampling depth of 3200 reads per sample. Additionally, for ease of comparison of this simplified data set, proportions were used to show compositional shifts with bar charts. Thirdly, we used the variance of OTUs across samples to filter out OTUs that did not vary across the samples. OTUs with variance greater than 1 x 10 ^-9^ were kept in in a highly-subsetted object, nb_pruned, containing just 29869 of the original 98580 OTUs at 1e-12 variance cut off, nb_pruned was used in further exploratory visualisations such as networks highlighting the discriminant taxa between samples. Finally, data were transformed to project the bacterial phylogeny annotated with host and soil sample metadata. To make this figure sufficiently uncluttered we removed OTUs not recorded more than 10 times in at least 20% of samples, counts were rarefied to an even sampling depth of 200 reads per sample. Samples were then merged by host and soil, and OTU’s agglomerated by family, this resulted in the most summarised data set nd_tree. Analysis scripts and data are available on request.
